# Erratum to: SuperTranscripts: a data driven reference for analysis and visualisation of transcriptomes

**DOI:** 10.1186/s13059-017-1303-2

**Published:** 2017-08-24

**Authors:** Nadia M. Davidson, Anthony D. K. Hawkins, Alicia Oshlack

**Affiliations:** 1 Murdoch Children’s Research Institute, Royal Children’s Hospital, Melbourne, Australia; 2 School of BioSciences, University of Melbourne, Melbourne, Australia

## Erratum

Upon publication of the original article [1], it was noted that data submitted by the authors was erroneously amended during proofing. The following section of the submitted manuscript was wrongfully amended in the Methods section, in the published article [1]:

The second line of the subsection “Read alignment” currently reads “--outSJfilterOverhangMin 12 12 12” and should instead be “--outSJfilterOverhangMin 12 12 12 12”.
