# Supplementary material for: SuperTranscripts: a data driven reference for analysis and visualisation of transcriptomes
Source: Genome Biol. 2017 Aug 4;18:148. doi: 10.1186/s13059-017-1284-1 (PMC5543425; doi:10.1186/s13059-017-1284-1)
Supplement: Additional file 1: — Contains supplementary figures and tables. (PDF 817 kb) [file 13059_2017_1284_MOESM1_ESM.pdf]

Additional File 1:
Supplementary Tables and Figures for:

SuperTranscripts: a data driven reference for analysis and visualisation of transcriptomes

Nadia M Davidson[1*], Anthony DK Hawkins[1], Alicia Oshlack[1,2*]

[1]Murdoch Childrens Research Institute, Royal Children's Hospital, Victoria, Australia
[2]School of BioSciences, University of Melbourne, Victoria, Australia

[*]Corresponding authors: alicia.oshlack@mcri.edu.au, nadia.davidson@mcri.edu.au

Contents:

Length of longest isoform compared to superTranscript:

Table S1

Figure S1

Lace software:

Figure S2

Variant calling in non-model organisms:

Table S2

Figure S3

Chicken superTranscriptome:

Figure S4

Figure S5

Applications to model organisms:

Table S3

Figure S6

Figure S7

Length of longest isoform compared to superTranscript:

The longest isoform of each gene is often used as a reference in studies of non-model organisms. It provides the same convenience as superTranscripts because there is only a single sequence per gene and tasks such as viewing reads in IGV or calling variants using GATK can be easily achieved. However, unlike superTranscripts, which contain the complete sequence of each gene, the longest isoform may miss sequence from alternative isoforms. Here we assess the extent that sequence is missed when the longest isoform is used rather than the superTranscript. We examined two different transcriptomes for human 1) the Ensembl annotation for hg19, where superTranscripts were constructed by concatenating exonic sequence and 2) An RNA-Seq sample from Genome in a Bottle which was de novo assembled with Trinity, then had transcripts reassembled into superTranscripts with Lace.

Table S1 – The length of various transcriptome references. For both the reference transcriptome and assembled transcriptome, the superTranscriptome was significantly more compact than the full transcriptome (which contains the sequence of every isoform), despite both references containing the same information content. Compared to superTranscripts, the longest isoform has 25% (Ensembl GRCh37.71) and 5% (Genome in a Bottle) less sequence on average and therefore less information content.

	Transcriptome (Mbp)	superTranscriptome (Mbp)	Longest Isoforms (Mbp)
Ensembl GRCh37.71	246	93	70
Assembled Genome in a Bottle	145	78	74

Figure S1 – Box plots showing the proportion of gene sequence that is missed if the longest isoform only is used as a reference, where proportion of each gene is as: (the length of the superTranscript – the length of longest isoform) / the length of the superTranscript. Genes are grouped according to how many transcripts they have. As expected, there is no difference for single transcript genes as the superTranscript is the longest isoform. The proportion of sequence missed tends to increase with the number of transcript in the gene. (A) Annotated human transcripts (B) assembled data. Note that here the superTranscript can occasionally be shorter than the longest isoform when a gene contains repeated sequence which is reduced to a single block by Lace.

A – Ensembl GRCh37.71

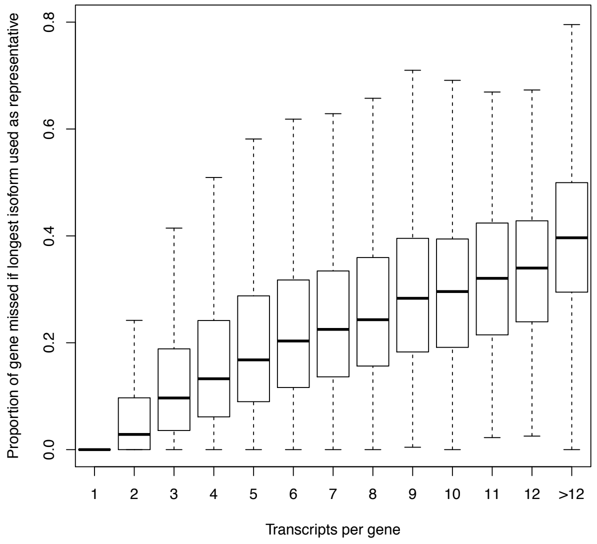

B –Assembled Genome in a Bottle

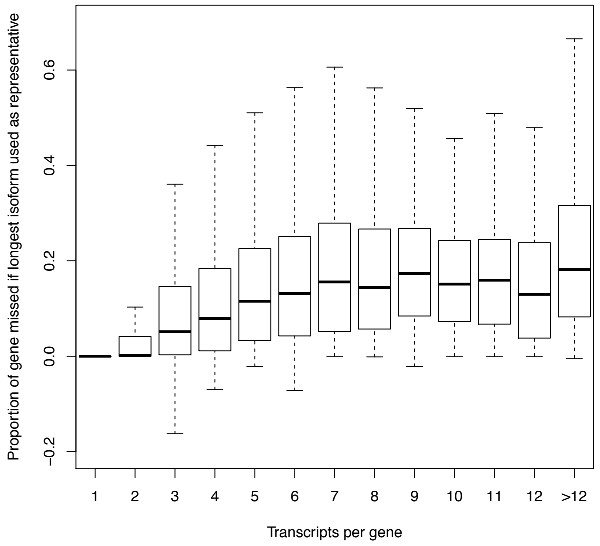

Lace software:

Figure S2 – An illustration of how the Lace software removes cycles in overlap graphs in order to create DAGs (directed acyclic graphs) which can be topologically sorted. The node in the cycle with the fewest bases (green) is repeated and the incoming and outgoing edges on that node are rerouted in order to break the cycle.

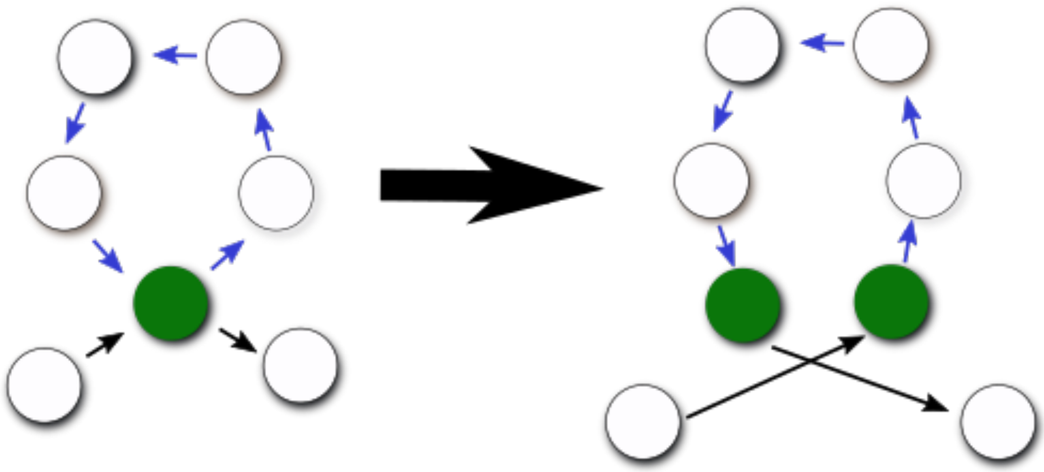

Variant calling in non-model organisms:

Table S2 – SNPs were called on RNA-seq generated from the Genome in a Bottle cell line using three different methods: 1) Genome approach - read alignment to the hg38 reference genome using STAR followed by GATK. 2) SuperTranscript approach - de novo assembly, followed by Lace, read alignment with STAR and finally GATK and 3) KisSplice. Below we show the number of SNPs reported after various filtering steps. For superTranscripts and KisSplice we also show the number of SNPs in common with the genome approach.

	1) Genome	2) SuperTranscripts		3) KisSplice	
		Total	Overlap with genome approach	Total	Overlap with genome approach
Variants called	162,890	87,736	N/A	33,252 paths (can have multiple variants)	N/A
After applying standard GATK filter + removing indels, homozygous SNPs and variants with <10 read coverage.	24,696	26,367	N/A	N/A	N/A
Variants where a unique position could be found in the genome:	24,696	24,477	16,708	28,447	12,801
Variants found in the high confidence call regions for Genome in a Bottle:	19,706	17,035	13,938	20,922	10,581
Variants overlapping Genome in a Bottle calls (true positives - TP)	15,925 (81%)	13,639 (80%)	12,690	11,269 (54%)	10,287
Excluding repeat regions:					
True positives	13,227	11,817	11,015	9,863	9,111
Total reported	13,857	13,082	11,268	17,217	9,151
Precision	95%	90%		57%	

N/A – Could not be assessed

Figure S3 – Calling variants from *de novo* assembled RNA-Seq. On the *de novo* assembled Genome in a Bottle dataset, we constructed superTranscripts with Lace and searched for variants using the GATK's Best Practices workflow. Shown is an example of two heterozygous SNPs that were detected. Both SNPs are common variants in an intron of LSM8. The variant on the left was missed due to low coverage when we ran GATK on reads that had been aligned to the hg38 reference.

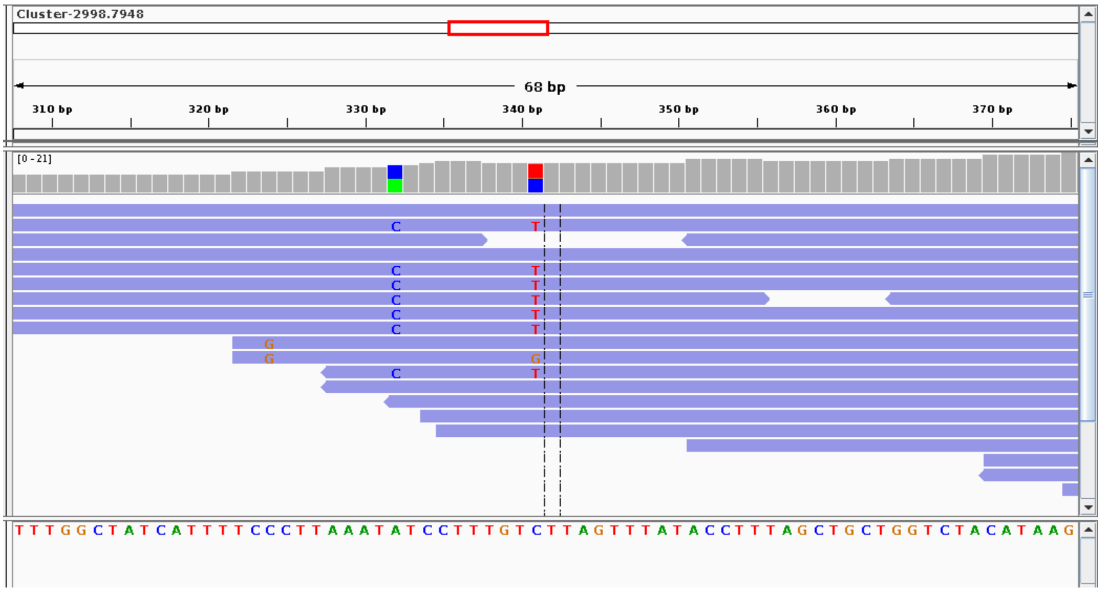

Chicken superTranscriptome:

Figure S4 - Annotation of the gene, C22orf39 in the chicken reference genome. A genomic gap of approximately 100bp can be seen in galGal4 (A). The superTranscript of this gene recovers the gap sequence (Figure 3A in the paper). The genomic gap is filled in galGal5 (B) and the full superTranscript sequence aligns, however the gene is not annotated in Ensembl.

A - galGal4

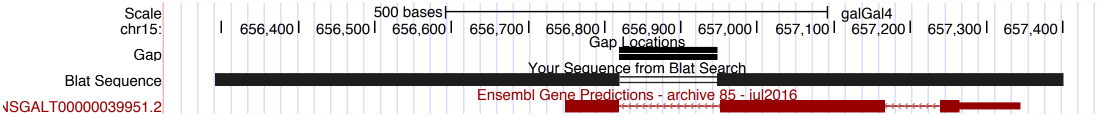

B - galGal5

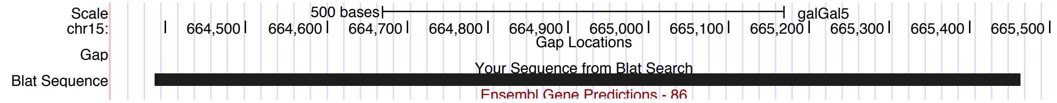

Figure S5 - The number of superTranscripts with partial sequence absent from the chicken reference genome: A) galGal4 and B) galGal5, by chromosome. In most instances, the superTranscript overlaps a known gap in the reference (dark histogram) or is positioned on a short poorly assembled fragment (labelled "Un").

A – galGal4

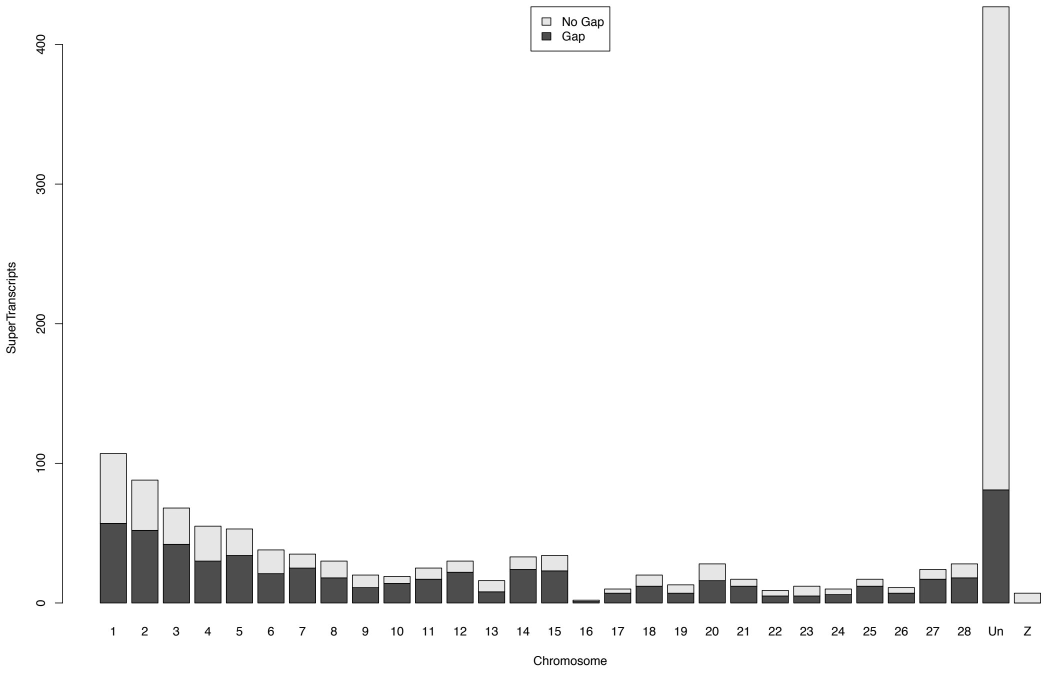

B – galGal5

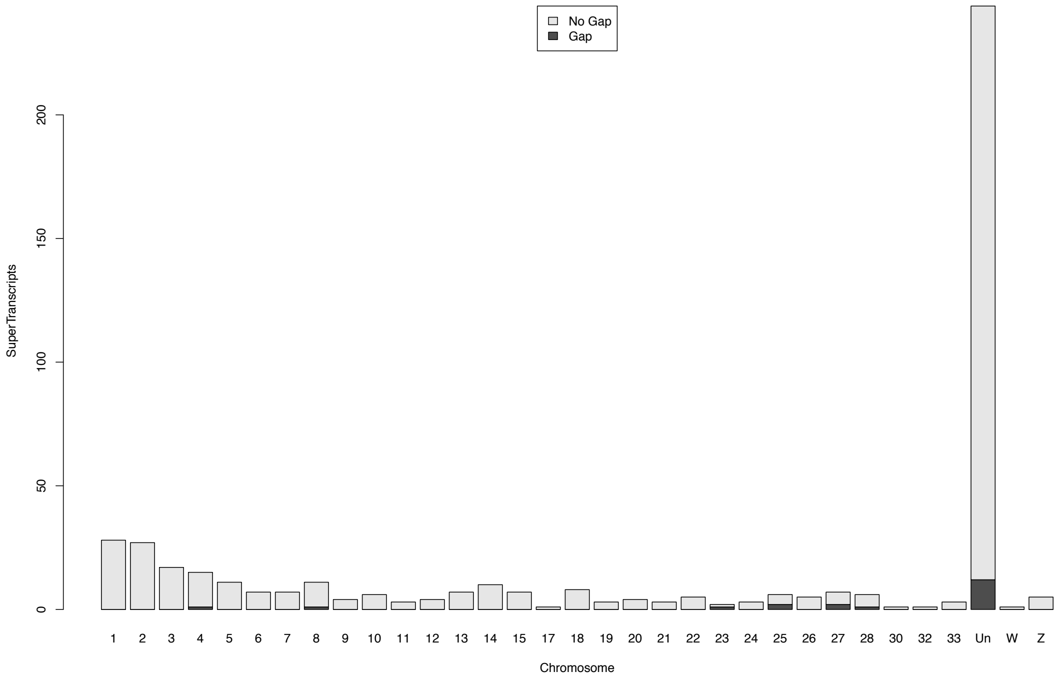

Applications to model organisms:

Table S3 - We used simulated human RNA-Seq reads to evaluate the use of superTranscripts compared with aligning to the human reference genome (see Soneson et al, 2016 for simulation details). Reads were mapped to either the genome or superTranscriptome (standard blocks) using STAR, then counted with featureCounts. The superTranscriptome had slightly more reads counted than the genome and substantially fewer reads split by a splice junction.

	Reads aligning (40 million simulated)	Average number of reads counted by Feature Counts per sample	Average reads spanning a splice junction
Genome	37,981,831	36,142,217	22,614,778
SuperTranscripts	37,947,640	36,832,471	10,410,788

Figure S6 – An IGV screenshot of the SuperTranscript constructed by Lace for ENSG00000108443 from the MCF-7 2013 PacBio dataset (http://www.pacb.com/blog/data-release-human-mcf-7-transcriptome/), after alignment back to the superTranscript using blat (Top). The transcript coverage for the same gene. (Bottom) This image is produced by Lace.

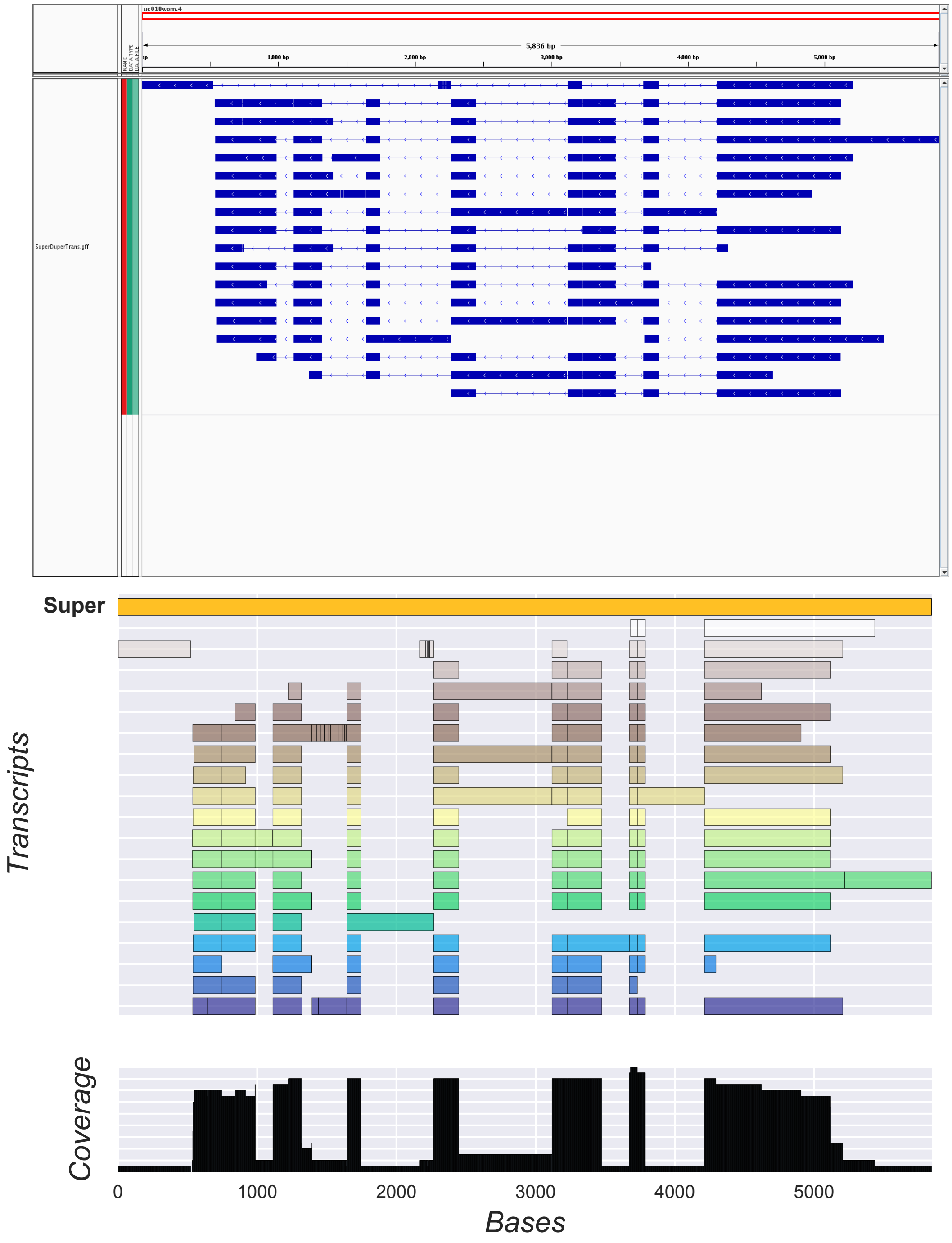

Figure S7 – Simulated human RNA-Seq suggests superTranscripts improve detection of differential isoform usage even when a reference genome is available. The simulated dataset included 1000 genes where differential isoform usage was simulated between two isoforms in a gene without changing the overall expression levels of the gene (see Soneson et al, 2016 for details). We used the default settings in DEXSeq to test for differential isoform usage on the featureCounts output for each of the three methods: a flattened genome annotation; standard blocks in the superTranscriptome; and dynamic blocks in the superTranscriptome. (A) A per gene q-value was calculated for every gene and compared to the simulated truth in order to build a Receiver Operator Curve (ROC). (B) A box plot of the counts per gene divided by the number of blocks per gene (counts per block) relative to the counts per block from the genome annotation. SuperTranscripts have more power to detect differential isoform usage because there are more counts per block.

A

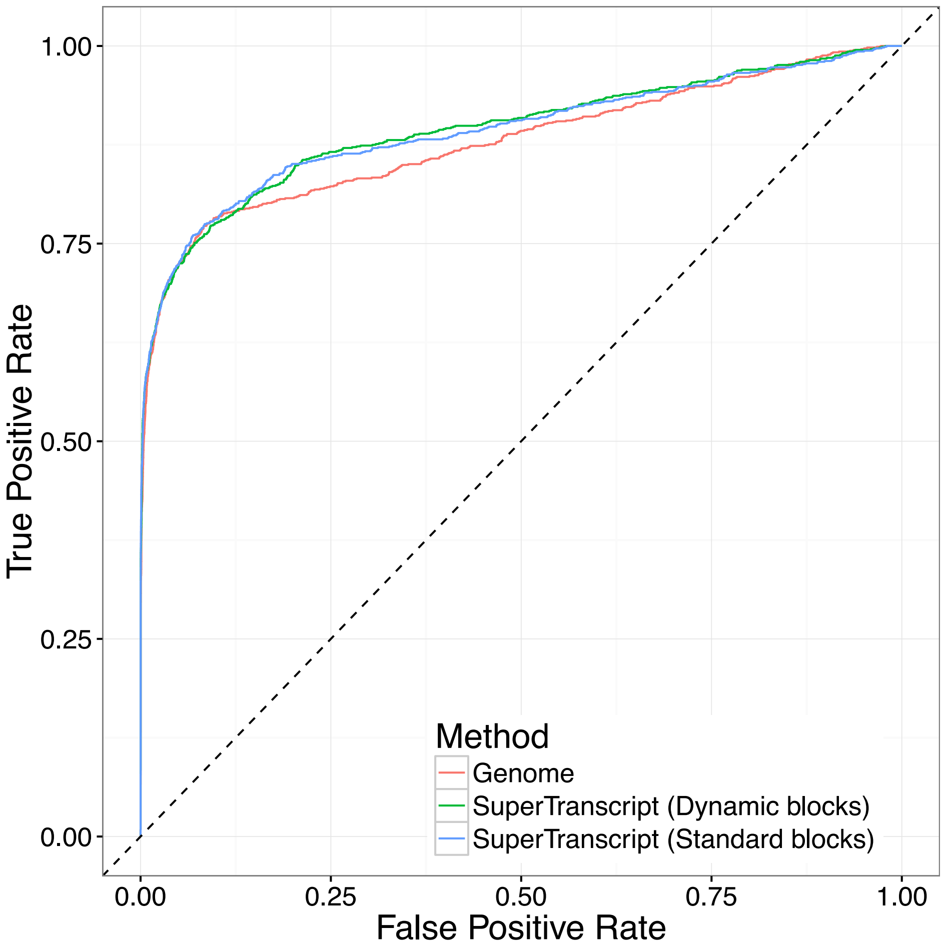

B

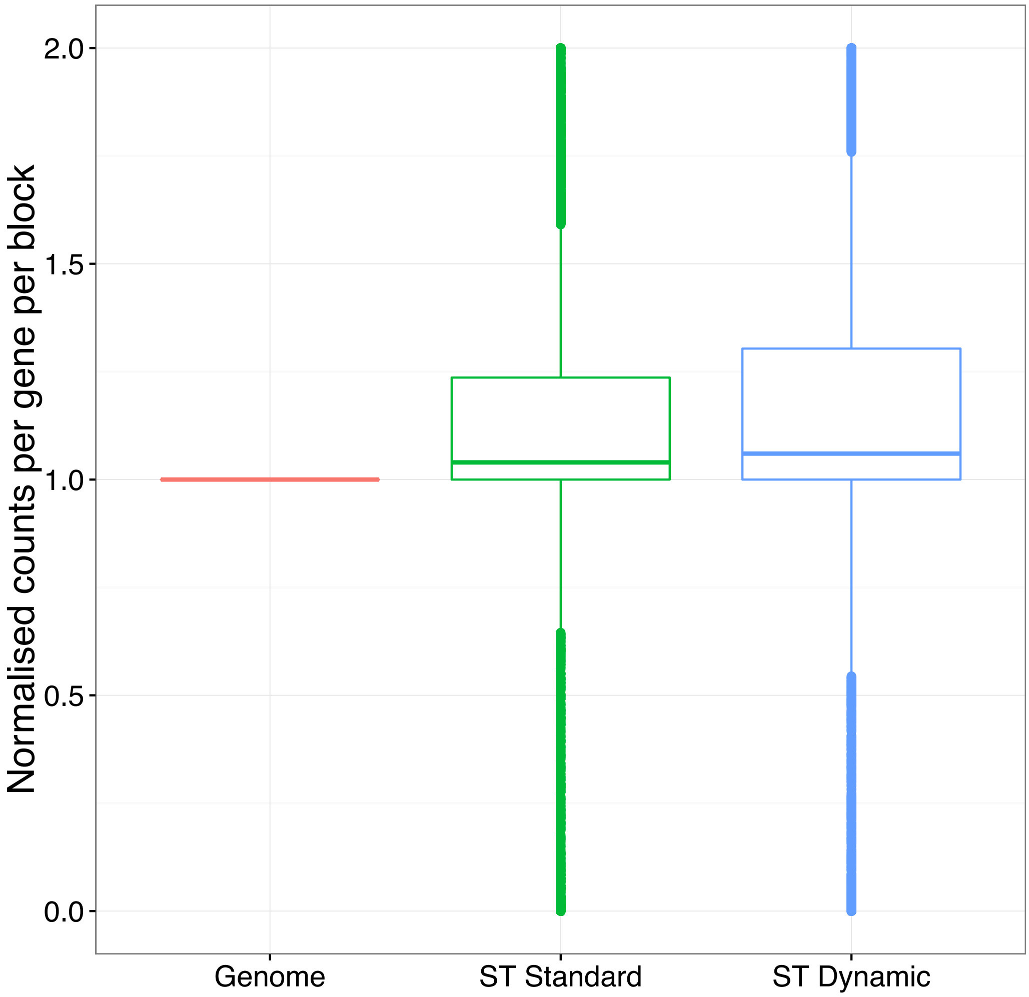

Normalised counts per gene per block
2.0
1.5
1.0
0.5
0.0
Genome
ST Standard
ST Dynamic
